# Model selection versus traditional hypothesis testing in circular statistics: a simulation study

**DOI:** 10.1242/bio.049866

**Published:** 2020-06-23

**Authors:** Lukas Landler, Graeme D. Ruxton, E. Pascal Malkemper

**Affiliations:** 1 Institute of Zoology, Department of Integrative Biology and Biodiversity Research, University of Natural Resources and Life Sciences Vienna, Gregor-Mendel-Strasse 33, A-1180 Vienna, Austria; 2 Research Institute of Molecular Pathology (IMP), Vienna Biocenter (VBC), Campus-Vienna-Biocenter 1, 1030 Vienna, Austria; 3 School of Biology, University of St Andrews, St Andrews KY16 9TH, UK; 4 Max Planck Research Group Neurobiology of Magnetoreception, Center of Advanced European Studies and Research (caesar), Ludwig-Erhard-Allee 2, Bonn 53175, Germany

**Keywords:** Circular statistics, AIC, Rayleigh test, Hermans-Rasson test

## Abstract

Many studies in biology involve data measured on a circular scale. Such data require different statistical treatment from those measured on linear scales. The most common statistical exploration of circular data involves testing the null hypothesis that the data show no aggregation and are instead uniformly distributed over the whole circle. The most common means of performing this type of investigation is with a Rayleigh test. An alternative might be to compare the fit of the uniform distribution model to alternative models. Such model-fitting approaches have become a standard technique with linear data, and their greater application to circular data has been recently advocated. Here we present simulation data that demonstrate that such model-based inference can offer very similar performance to the best traditional tests, but only if adjustment is made in order to control type I error rate.

## Abstract (precis)

**Summary:** Model selection-based statistics is a promising new avenue for analyzing circular data. We describe a procedure to control the type I error rate.

## INTRODUCTION

Circular data are common in natural sciences, ranging from directional (e.g. animal orientation) to time-dependent data (e.g. annual cycles and circadian rhythms) and treatment of such data has been reviewed in several monographs (Batschelet, 1981; Fisher, 1995; Ley and Verdebout, 2017; Mardia and Jupp, 2009; Pewsey et al., 2013). The most common statistical exploration of circular data involves testing the null hypothesis that the data are uniformly distributed across all possible values around the circle (versus some form of concentration). Overwhelmingly the most common approach to testing this null hypothesis is the Rayleigh test (Rayleigh, 1880). Although it was one of the earliest circular statistical techniques, the Rayleigh test is the most powerful test for detecting some unimodal departures (e.g. Von Mises and projected normal alternative) from circular uniformity (Bhattacharyya and Johnson, 1969; Landler et al., 2018; Mardia and Jupp, 2009). Other tests that are often used in circular statistics and are known to detect unimodal and multimodal departures from uniformity include the Rao's spacing test, the Watson test and the Kuiper's test (see Batschelet, 1981 for an overview). However, more computationally-demanding simulation-based approaches have been developed more recently (Pewsey et al., 2013). One such test is the Hermans-Rasson test, which has high statistical power over a broad range of situations and is considerably more powerful than the Rayleigh test for certain multimodally clustered distributions (Landler et al., 2019b). In addition, simulation approaches have also been proven useful to correct for type I error rate inflation due to rounded data (Landler et al., 2019a).

For linear data, there has been a broadening of statistical approaches over the last 20 years, with reduced emphasis on null-hypothesis testing and increased use of Bayesian and model-selection techniques. Such statistical approaches could also be applied to circular data, where null-hypothesis testing remains the dominant statistical approach. Recently, Fitak and Johnsen (2017) presented how model-based approaches can be used for circular data. Their approach involved fitting a suite of ten potential models to a sample of circular data and then using the Akaike Information Criterion (AIC; Aho et al., 2014; Akaike, 1973) to compare how well the data fitted the different models. The power of this methodology lies in not just identifying if the data is concentrated in some way (i.e. appears non-uniform) but describing the relative likelihood of different underlying processes generating different types of concentration. An example of the application of this approach is a recent study of the orientation of shorebirds in response to magnetic and visual cues (Vanni et al., 2017). The authors first used a Rayleigh test to establish if the null-hypothesis of uniformity can be rejected. In situations where it appeared that there was solid evidence to reject uniformity, the authors then used a model-based approach to compare the relative support for a unimodal concentration of data (suggesting that birds integrated conflicting magnetic and visual cues) or a bimodal situation (suggesting different birds relied on different cues). In order to exemplify the potential use of the approach described in here, we use a pigeon orientation data set available in the R package ‘circular’ (see Materials and Methods). The data are from Gagliardo et al. (2008). In this study, the authors tested the hypothesis that homing pigeons use magnetic and/or olfactory cues for homing. They accomplish this by performing anatomical lesions, where they either sectioned the olfactory nerve, the trigeminal nerve (which is thought to transmit magnetic information) or left both nerves intact. In their analysis, they concluded that only the olfactory nerve lesion impaired the pigeon homing performance and therefore magnetic map cues are not necessary for pigeon navigational abilities.

Our interest here is in exploring to what extent the model-based approach could be extended to encompass the role of the Rayleigh test, since circular uniformity is simply another model to which the sample of data can be applied. More generally, we want to offer advice to those interested in using model-based approaches to samples of circular data as to whether they can streamline their analysis or whether they should retain a traditional test of the null hypothesis of circular uniformity as well as their model-fitting exercise.

## RESULTS AND DISCUSSION

**Fig. 1.**
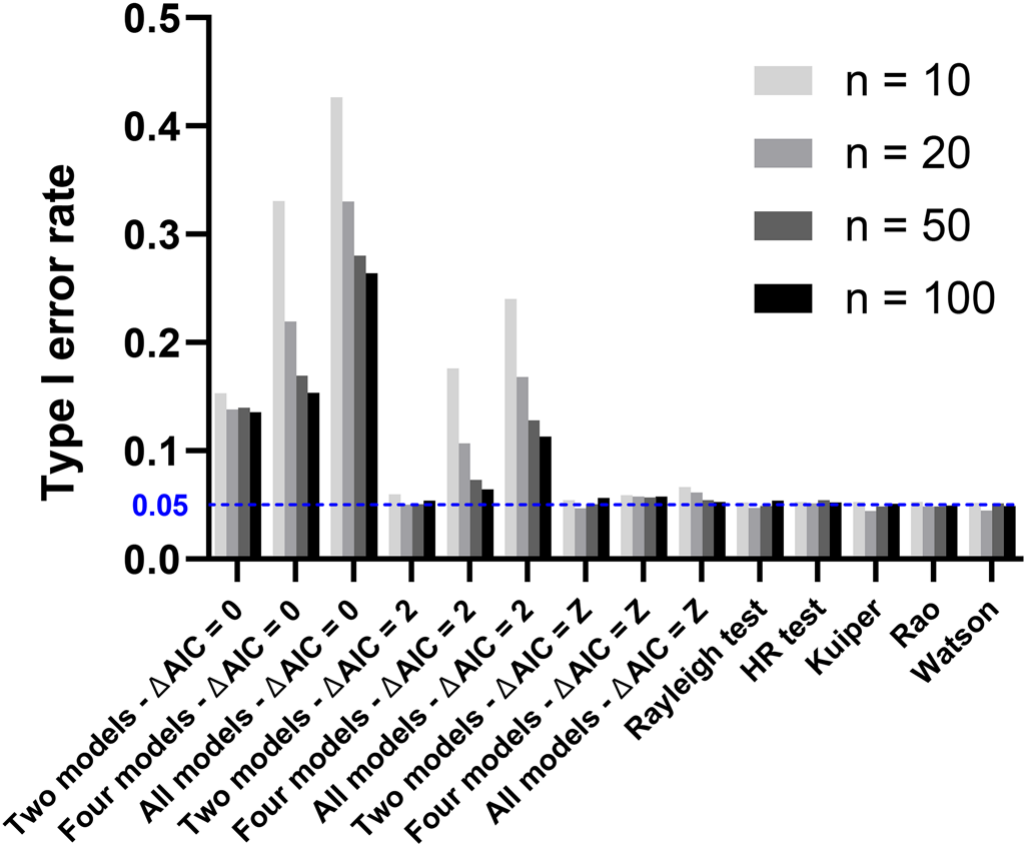
**Type I error rate of all tests applied.** For the AIC based model fitting we used three different delta AIC criteria: 0, 2, Z. In the AIC approach we either compared two, four or ten (all) models. Sample sizes are given in the legend. The dashed line represents the 0.05 nominal significance level.

Our results show that model-fitting based results have to be treated with caution when the false discovery rate is not controlled for. When using delta AIC=0 as a cut-off, up to 40% of instances where we tested a sample from a truly circular uniform distribution, rejection of that model was suggested by the AIC model fitting (Fig. 1). Such type I error rate decreased with sample size but was controlled for when we used the cut-off value Z (see Table S1), which we derived by simulation (Fig. 1). The delta AIC=2 cut-off, which was used by Fitak and Johnsen (2017), resulted in controlled type I error only in cases where two models were tested against each other (in these instances Z is very close to 2; see Table S1). In comparison, the Rayleigh, Rao, Kuiper’s and Watson test as well as the HR test reliably showed type I error rates close to the nominal value (0.05). This suggests that a model-fitting approach shows comparable performance to traditional tests of the null hypothesis of circular uniformity if the suite of models tested is limited to two (uniformity and a unimodal concentration). Larger suites can be accommodated but the criterion for rejection of the null hypothesis has to be tuned so as to avoid substantial inflation of the type I error rate. This is true even for large sample sizes.

**Fig. 2.**
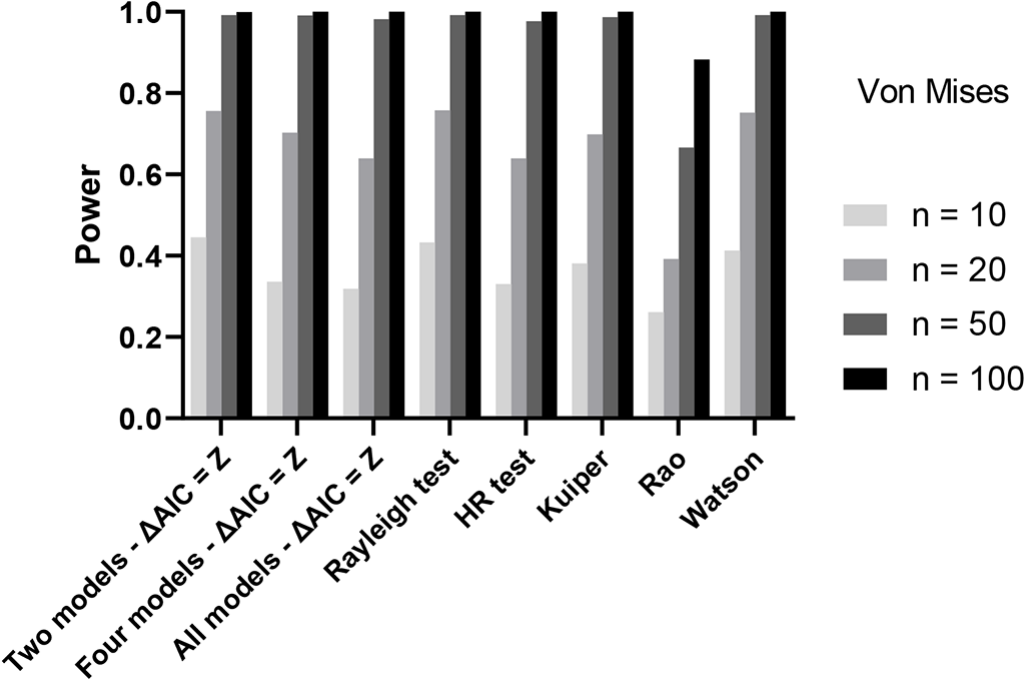
**Power of all tests with controlled false positive rate using a Von Mises distribution.** In the AIC approach we either compared two, four or ten (all) models, using Z as the cut-off. Sample sizes are given in the legend.

However, how powerful is the model fitting approach when using distributions that are non-uniform? We compared power in detecting a Von Mises distribution of all approaches with controlled false discovery rate, the alpha-corrected AIC approach, Rayleigh, Kuiper's, Watson, Rao and HR tests (Fig. 2). Here we saw that the AIC approach had broadly equivalent power to the Rayleigh, Kuiper's, Watson and HR tests and better power than the Rao test. Indeed, when only two models (circular uniform and Von Mises) were used, our version of the AIC approach even outperformed the conventional tests (Fig. 2). The more models were added in the AIC calculation, the lower the power. However, even when all ten models were included, power was comparable to the HR, Kuiper's and Watson test, and only slightly lower than the Rayleigh test.

**Fig. 3.**
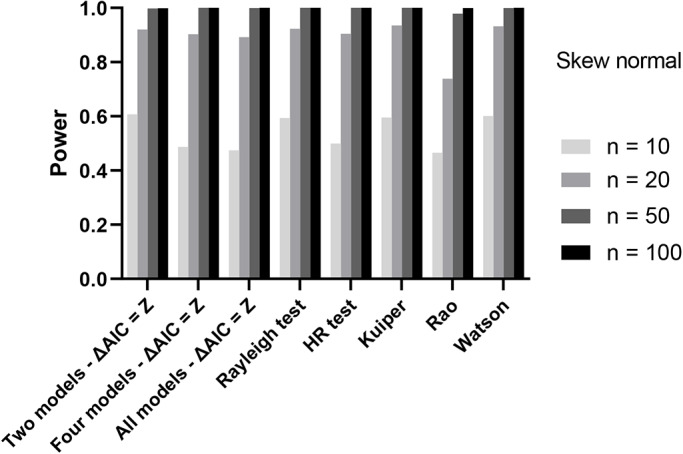
**Power of all tests with controlled false positive rate, testing a wrapped skew normal distribution.** In the AIC approach we either compared two, four or ten (all) models, using Z as the cut-off. Sample sizes are given in the legend.

**Fig. 4.**
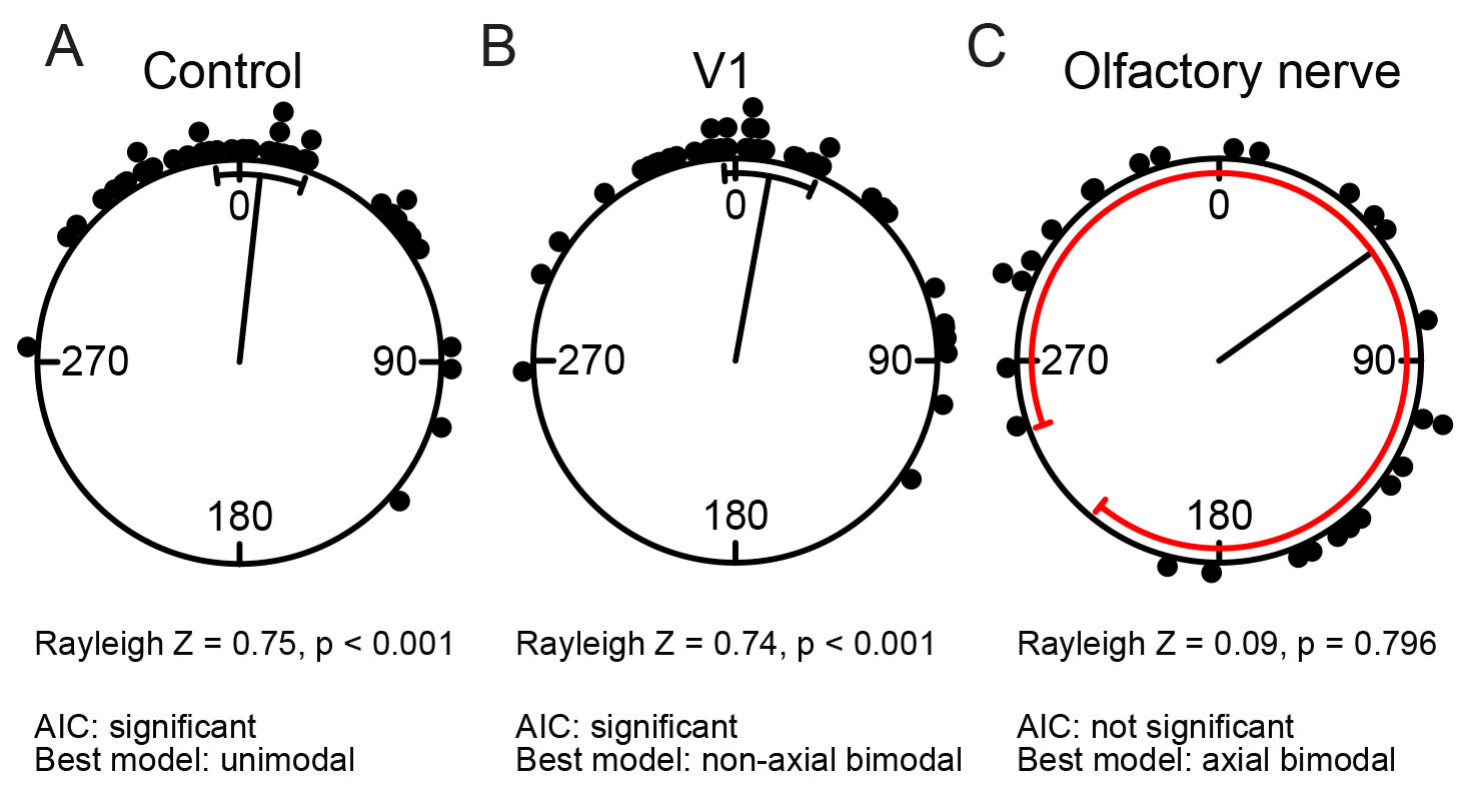
**Example analysis of pigeon homing data, showing three groups: (A) control pigeon (B) bilateral section of the ophthalmic branch (V1) of the trigeminal nerve (C) bilateral section of the olfactory nerve (on).** Data from Gagliardo et al. (2008).

Results for the asymmetrical unimodal distribution, the wrapped skew normal, showed similar behavior of the AIC approach, with power levels comparable between the three approaches (Fig. 3). Similarly, when we analyzed the AIC approach for the pigeon data, the general results were comparable (Fig. 4). Both distributions that were significant using the Rayleigh test (controls and v1) are significant using the AIC approach (controlling type I error using Z); the one distribution not significant using the Rayleigh test (on) also failed to show a significance using the AIC model approach. However, arguably, the AIC approach provides more information on the distributions; it indicates for example that the best model in the case of olfactory deprived pigeons may be axial instead of unimodal. This potentially indicates that pigeons without intact olfactory nerves still show a defined orientation behavior. Further experimentation might be needed to differentiate between an axial (relative to home) versus uniform (random) alternative.

We conclude that researchers keen to take a model-fitting approach to samples of circular data can potentially streamline their analysis by omitting a traditional test of the null hypothesis of circular uniformity and subsuming its function into their model fitting approach. However, if taking this approach, they must take care to avoid inflation of type I error rate. We believe the approach we take here offers a generally applicable template for that. Using such an approach can potentially increase the information a researcher can get regarding the resulting distribution while still maintaining comparable power as well as control of type I error. This is also shown in the presented pigeon example, where we might get a more informed view on the non-significant part of the results that allows us to plan follow up experiments.

However, for balance, we should also note that we do not have evidence that this approach will offer substantial statistical power benefits over the traditional tests. The motivation for subsuming the function of these tests into the model fitting framework instead come from brevity of presentation and philosophical uniformity within an analysis. An author's motivation for retaining the traditional tests should come from their well-studied performance and from commonality with previous works. The relative weighing of these issues will vary between investigators and investigations, but we hope this paper offers researchers the evidence-base for making a good decision. Further, we hope that this paper leads to a wider uptake of the AIC- and BIC-based approaches in circular statistics. More research is clearly needed to expand the availability of model fitting approaches in circular statistics to levels currently available in linear statistics.

## MATERIALS AND METHODS

Null-hypotheses tests of circular uniformity such as the Rayleigh test return a *P*-value between 0 and 1, which is the probability of seeing data similar to or more clustered than the observed sample if the underlying process was actually one of circular uniformity. By convention *P*-values less than some pre-specified level (often 0.05) are considered as evidence for rejecting the null hypothesis, whereas larger values are considered as evidence for not rejecting. The performance of such a test is evaluated in terms of its control of type I error rate and its statistical power (see R code published alongside the manuscript for all necessary code to re-run the analyses shown here). In terms of control of type I error rate, the test behaves well if presented with data from an underlying uniform distribution it generates a *P*-value below 0.05 (erroneously suggesting evidence against uniformity) on close to 5% of occasions. Statistical power is the probability that when presented with data from a specified non-uniform distribution the model generated a *P*-value below 0.05 (correctly suggesting evidence for rejecting the null hypothesis of uniformity).

We wanted to be able to evaluate equivalents to type I error rate and statistical power from a model-comparison approach. First, we must identify a suite of models. We used subsets of the models available in the R package (CircMLE) introduced by Fitak and Johnsen (2017), and originally proposed by Schnute and Groot (1992). In the first approach we only included the random model of circular uniformity (denoted M1 in the package documentation) and a Von Mises alternative (M2A). In the second approach we added two more alternative unimodal distributions (M2B, M2C), and in the last approach we used all unimodal and bimodal models available in the package in addition to the uniform distribution (ten in total, see Fitak and Johnsen 2017, for details).

As a measure of the evidence for or against circular uniformity, we used the AIC difference between the model of circular uniformity (M1) and the best model (i.e. that with the lowest associated AIC value) estimated from the function circ_mle() (Fitak and Johnsen, 2017). In order to mimic null-hypothesis testing we must specify a specific minimum difference in AIC (denoted delta AIC) that we take to imply that hypothesis of uniformity would be justified. We explored three different cut-offs for delta AIC: 0, 2 and Z. That is, with a cut-off of zero, if any of the other models considered had a lower AIC than M1, then this was taken as evidence for rejecting the null hypothesis of circular uniformity. With a cut-off of 2 we required that at least one other model in the suite being compared had an AIC value at least 2 units lower than M1 for uniformity to be rejected.

We derived a calculated critical value Z, separately for each sample size and suite of models, which preserved the type I error rate at the specified level. That is, we generated 10,000 samples of a circular uniform distribution and determined the value of Z that suggested erroneous rejection of uniformity in exactly 5% of cases.

We estimated type I error of the AIC-model based approach by generating 10,000 samples of a random distribution and calculating the fraction of samples resulting in delta AIC larger than 0, 2 and Z (see above). Of course, by definition the type I error rate for a cut-off of Z will be 0.05. In addition we tested the type I error for the Rayleigh test, using the function rayleigh.test(), the Watson test using the function watson.test(), the Kuiper test using the function kuiper.test(), the Rao spacing test using the function rao.spacing.test() (Agostinelli and Lund, 2013) and the Hermans-Rasson (HR) test (Landler et al., 2019b), as the fraction of significant tests (*P*<0.05) out of the same 10,000 random samples. Distributions were generated using the function rcircmix() (Oliveira Pérez et al., 2014) and the samples sizes 10, 20, 50, 100. The parameter ‘model’ was set to 1 for the circular uniform distribution.

For power estimation we generated the distributions using again the function rcircmix() (samples sizes: 10, 20, 50, 100), the model parameter was set to 2 for Von Mises and a wrapped skew normal distribution was generated using the parameters κ=30 and con=2. We restricted our analyses to unimodal alternatives, which is arguably the predominant type of distributions in orientation biology. However, in order to test a non-symmetric alternative, in addition to the well-studied Von Mises distribution, the wrapped skew normal distribution was added (see Fig. S1 for examples of the distributions used in this study).

We estimated statistical power as the fraction of 10,000 samples that resulted in delta AIC (defined as above) larger than Z (cut-offs of 0 and 2 were not used because of inflated type I error rates, see Fig. 1). The power of the Rayleigh test and HR Test was defined as the fraction of tests that resulted in *P*<0.05. Notice that power here is defined not as the probability of correctly identifying the particular non-random model that underlies the data – but correctly rejecting the null hypothesis of uniformity.

In addition to the simulation approach, we used the AIC model based approach as described above on an example pigeon data set available from the R package circular (Agostinelli and Lund, 2013). The pigeon data includes initial orientation of three groups (control pigeons; v1: bilateral section of the ophthalmic branch of the trigeminal nerve; on: bilateral section of the olfactory nerve) of homing pigeons derived from the experiment by Gagliardo et al. (2008). In addition, we performed a Rayleigh test for the same data, to exemplify the similarities and differences between the approaches.

## Supplementary Material

Supplementary information
