# Supplementary material for: Model selection versus traditional hypothesis testing in circular statistics: a simulation study
Source: Biol Open. 2020 Jun 23;9(6):bio049866. doi: 10.1242/bio.049866 (PMC7327993; doi:10.1242/bio.049866)
Supplement: Supplementary information [file biolopen-9-049866-s1.pdf]

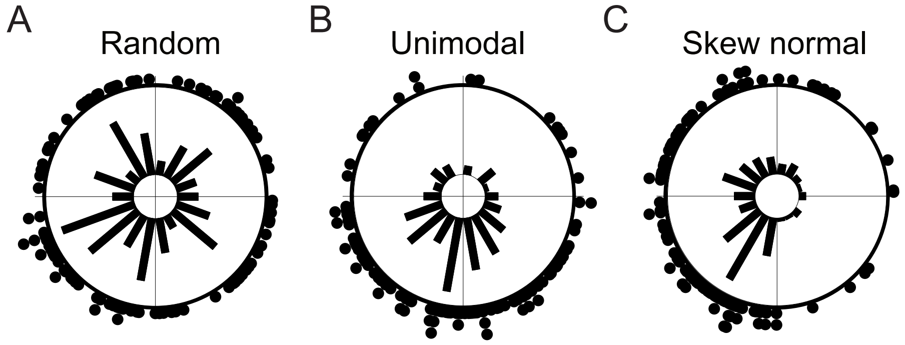

Biology Open • Supplementary information

Table S1: Calculated Z-values for the simulations conducted.

	Number of alternative models used		
Sample size	2	4	10
10	2.207	4.898	5.736
20	2.174	3.709	4.889
50	2.007	2.528	4.007
100	1.903	2.249	3.738

Dataset 1

Click here to Download Dataset 1

Dataset 2

Click here to Download Dataset 2

Dataset 3

Click here to Download Dataset 3
